# Comparison of the nasopharynx microbiome between influenza and non‐influenza cases of severe acute respiratory infections: A pilot study

**DOI:** 10.1002/hsr2.47

**Published:** 2018-05-09

**Authors:** Luiz Gustavo dos Anjos Borges, Adriana Giongo, Leandro de Mattos Pereira, Fernanda J. Trindade, Tatiana Schäffer Gregianini, Fabrício Souza Campos, Elodie Ghedin, Ana Beatriz Gorini da Veiga

**Affiliations:** ^1^ Laboratório de Biologia Molecular, Programa de Pós‐Graduação em Patologia Universidade Federal de Ciências da Saúde de Porto Alegre (UFCSPA) Porto Alegre RS Brazil; ^2^ Department of Microbiology Icahn School of Medicine at Mount Sinai New York NY USA; ^3^ Instituto do Petróleo e dos Recursos Naturais (IPR) Pontifícia Universidade Católica do Rio Grande do Sul (PUCRS) Porto Alegre RS Brazil; ^4^ Faculdade de Biociências Pontifícia Universidade Católica do Rio Grande do Sul (PUCRS) Porto Alegre RS Brazil; ^5^ Laboratório Central de Saúde Pública da Secretaria de Saúde do Estado do Rio Grande do Sul (LACEN/SES‐RS) Porto Alegre RS Brazil; ^6^ College of Veterinary Medicine and Agronomy University of Brasília, Darcy Ribeiro University Campus, ICC Asa Norte, CEP 70.910-970 Brasília DF Brazil; ^7^ Center for Genomics and Systems Biology, Department of Biology New York University New York NY USA; ^8^ Department of Epidemiology, College of Global Public Health New York University New York NY USA

**Keywords:** coinfection, influenza A virus, microbiome, respiratory disease, severe acute respiratory infection, upper respiratory tract

## Abstract

**Aims:**

Influenza A virus (IAV) can cause severe acute respiratory infection (SARI), and disease outcome may be associated with changes in the microbiome of the nasopharynx. This is a pilot study to characterize the microbiome of the nasopharynx in patients hospitalized with SARI, infected and not infected by IAV.

**Methods and Results:**

Using target sequencing of the 16S rRNA gene, we assessed the bacterial community of nasopharyngeal aspirate samples and compared the microbiome of patients infected with IAV with the microbiome of patients who were negative for IAV. We observed differences in the relative abundance of Proteobacteria and Firmicutes between SARI patients, with *Streptococcus* being enriched and *Pseudomonas* underrepresented in IAV patients compared with patients who were not infected with IAV.

**Conclusion:**

*Pseudomonas* taxon seems to be in high frequency on the nasopharynx of SARI patients with non‐IAV infection and might present a negative association with *Streptococcus* taxon. Microbial profile appears to be different between SARI patients infected or not infected with IAV.

**Abbreviations**
IAV: influenza A virus
OTUs: operational taxonomic units
PCoA: principal coordinate analysis
SA: sialic acid
SARI: severe acute respiratory infection
URT: upper respiratory tract

## 1 INTRODUCTION

Influenza A virus (IAV) infection is among the most common and major causes of human respiratory infection, presenting high morbidity and mortality worldwide, with hundreds of thousands of hospitalizations and deaths every year.^1^ Hospitalized patients with influenza disease exhibit a variety of nonspecific influenza‐like symptoms that may also be observed in patients with other respiratory infections. Hospitalization fatality risk is the probability of death associated with H1N1pdm09 cases in a cohort of individuals that required hospitalization for medical reasons.^2^ While the influenza‐like symptoms of flu patients are commonly considered as a measure of disease severity, and determine whether the patients suffer from a severe acute respiratory infection (SARI), hospitalization fatality risk during influenza virus infection has been underestimated.^2^ Thus, the addition of other measures that could impact severity—such as the microbiome—should be explored.

The microbiome could be defined as the collective genome of the microorganisms that reside in an environment niche.^3^, ^4^ Studies of the human microbiome have shown a remarkable diversity of microbes that occupy different habitats of the human body to establish a microbial community.^5^, ^6^ These microbial communities seem to be structurally stable over time, and this stability of the microbiome composition has been associated with specific behaviors of individuals, such as observed on healthy smokers,^7^ as well as to health condition of individuals, such as observed on patients with cystic fibrosis^8^ or infectious disease.^9^

The respiratory tract has been widely studied to understand the dynamics of respiratory infections.^10^ While lung samples are not easily accessible, nasal and oral samples have been used for investigating and identifying microorganisms responsible for lung infection.^10^, ^11^ Recently, comparative studies have shown that the bronchoalveolar microbiota may be better represented by a composition of oral and nasal microbiomes.^11^ However, IAV H1N1 subtype is a respiratory virus, and its transmission typically comprises airway introduction and success infection of the upper respiratory tract (URT).^12^

The microbiome of the URT is susceptible to disruption by pathogens. Influenza A virus infection, for example, has been shown to modify the community structure of the microbiome^13^ and to lead to the outgrowth of pathogenic bacteria.^14^ Proteobacteria, Firmicutes, Bacteroidetes, Actinobacteria, and Fusobacteria are common phyla found in variable proportions in the URT of healthy individuals.^15^ Even pathogenic bacteria can be present at low abundance in established communities.^5^ A specific ecological perturbation can, however, change the bacterial community structure, leading to local or systemic infection by both bacterial and viral pathogens,^16^ resulting in poor disease outcome for the patients. For example, commensal bacteria like *Streptococcus pneumoniae* can establish a mutually beneficial relationship with influenza virus.^17^ Studies suggest that a prior IAV infection could enhance the transmission of *S*. *pneumoniae*,^18^ which in turn, could modulate the innate immune response of the host in favor of IAV or even secrete proteases that could activate the viral hemagglutinin.^17^

Not all respiratory infections (viral or bacterial), however, are likely to modify the microbiome in the same manner, and this could have repercussions on disease severity and outcome for the patients. Major changes have been observed in the bacterial community of the URT of patients after viral infection.^14^ Once the patient is hospitalized because of SARI, changes in the microbiome could be more specific, and consequently, it may be possible to differentiate groups of patients with similar symptoms based on their URT microbiome. In a pilot study, Langevin et al^9^ suggested that the microbiota could be used for clinical management, as it appears that specific microbial signatures allow distinguishing between severe and mild influenza in children. Aiming to better understand the role that the microbiome of the nasopharynx plays during respiratory infection, we conducted this pilot study to determine whether the bacterial community in the URT of hospitalized patients with SARI has a different profile in cases of IAV infection, compared with other SARI cases.

## 2 MATERIALS AND METHODS

### 2.1 Ethics statement

The Research Board and Ethics Committee of Federal University of Health Sciences of Porto Alegre approved the present study (Ethics Statement no. 1774/12). Patients who were enrolled in the study were informed that their samples and the health‐related data collected would be used for disease diagnosis, clinical treatment, and epidemiological surveillance and that the data could be further used for scientific research. Patients were given the opportunity to refuse, and only data from patients who agreed with these terms were included in the study. All data were analyzed and reported anonymously and kept confidential. The authors did not have access to identifiers of research subjects other than clinical data, sex, age, and pregnancy and vaccination status.

### 2.2 Biological samples

Nasopharyngeal aspirates from 12 enrolled donors were obtained from a biorepository of the Central Laboratory of Public Health of Rio Grande do Sul. All samples were collected in hospital units of Rio Grande do Sul, Brazil, from January to September of 2012, from patients hospitalized with SARI and suspected of being infected with IAV. All samples had been screened at the Central Laboratory of Public Health of Rio Grande do Sul for other respiratory viruses by indirect immunofluorescence (IFA) using the Light Diagnostic Respiratory Panel I Viral Screening and Identification IFA Kit (catalogue no. 3105, Millipore), with monoclonal antibodies for respiratory syncytial virus, adenovirus, *Human metapneumovirus*, and *Human parainfluenza virus* 1, 2, and 3 according to the manufacturer's instructions.

Samples were considered eligible when medical records indicated no smoking behavior,^7^ no previous vaccine for IAV H1N1pdm09/H3N2,^19^ no comorbidities such as chronic pneumopathy or chronic heart disease, nonchronic viral diseases such as hepatitis C or HIV infection, and negative for other respiratory viruses.

### 2.3 RNA extraction and detection of IAV

RNA was extracted from samples using the QIAamp Viral RNA Mini Kit (Qiagen), according to the manufacturer's instructions. RNA quantity and purity were evaluated on the basis of absorbance (A_260_/A_280_ ratio) using NanoDrop ND‐1000 (Thermo Fisher), and the integrity of RNA extracted was verified using RNaseP (human RNase P gene) primer and probe as reverse transcription–polymerase chain reaction internal controls. Following the standard CDC protocol,^20^ each sample was subjected to reverse transcription–real‐time polymerase chain reaction by using primers and probe sets specific for detection of influenza A(H1N1)pdm09 and influenza A/H3. All internal positive and negative controls were included on reactions, as described on the cited protocol. Briefly, reactions using SuperScript III Platinum One‐Step Quantitative Kit (catalogue no. 11745‐100, Invitrogen) containing 0.5 μL of SSIII Platinum Taq Mix, 1μM of each primer, 0.25μM of probe, 12.5 μL of 2X Master Mix, 5 μL of RNA sample, and water to a final volume of 20 μL were performed in an Applied Biosystems 7500 Real‐Time PCR System (Thermo Fisher) following 50°C for 30 minutes, 95°C for 2 minutes, and 45 cycles at 95°C for 15 seconds and 55°C for 35 seconds.

### 2.4 High‐throughput sequencing and analysis

DNA was extracted from samples using QIAamp DNA Mini Kit (catalogue no./ID 51304, Qiagen) according to the manufacturer's instructions. DNA quantity was evaluated on the basis of fluorometric quantitation assay using Qubit dsDNA HS (Thermo Fisher), and the purity was evaluated on the basis of absorbance (A_260_/A_280_ ratio) using NanoDrop ND‐1000 (Thermo Fisher). The set of primers 515F (5′ GTGCCAGCMGCCGCGGTAA 3′) and 806R (5′ GGACTACVSGGGTATCTAAT 3′) was used to amplify an approximately 291 bp fragment from the V4 hypervariable region of the prokaryotic 16S rRNA gene.^21^ The polymerase chain reaction amplicons were purified using Agencourt AMPure Beads (catalogue no. A63880, Beckman Coulter), and 100 ng of purified DNA was used for Ion Plus Fragment Library construction (catalogue no. 4471252, Thermo Fisher), following the manufacturer's instructions. A negative sample control was not included in the sequencing.^22^ Each sample was barcoded and sequenced in a multiplexed PGM run (20 barcoded samples per run). Sequencing was conducted on an Ion PGM System (Thermo Fisher) using an Ion 316 chip, following the manufacturer's instructions. Sequencing data were deposited in the Sequence Read Archive of the National Center for Biotechnology Information, under sequence read archive accession number SRP073009.

All 16S rRNA gene reads produced by high‐throughput sequencing were subjected to quality control to retain sequences with a minimum length of 100 bp and were trimmed to remove low‐quality bases (minimum Phred score of 30) using PRINSEQ.^23^ Also, duplicated sequences were identified and sorted by decreasing read abundance and then filtered to exclude singletons, using USEARCH v7.0.1090.^24^ Clusters were assembled using a minimum identity of 99%, and chimeras were removed using the RDP reference database.^25^ Taxonomic assignment was obtained using QIIME v1.8.0,^26^ and operational taxonomic units (OTUs) were selected on the basis of 97% sequence similarity. Taxonomic data were generated through the classification algorithm using the 97% OTUs version of GreenGenes 13.8.^27^ The default parameters of QIIME v1.8.0 were used for the alignment of OTUs (pyNAST) and to generate phylogenies (FastTree). Rarefactions of the OTU table were performed on 10 steps of 500 sequences of subsampling for a maximum depth of 5000 sequences. Alpha diversity metrics were calculated using QIIME v1.8.0. Multiple rarefactions were performed for Chao1 (species richness), Shannon (the entropic information of the abundances of observed OTUs), Simpson_e (evenness), and Equitability. Beta diversity analysis was calculated using unweighted UniFrac. The principal coordinate analysis (PCoA) was generated to observe differences between groups, and the results were visualized using EMPeror software.^28^

### 2.5 Statistical analysis

Statistical analyses were done using SPSS 20.0 (IBM, USA). Data were presented as relative frequency or median and interquartile ranges. The Mann‐Whitney *U* test was used to compare the diversity between groups. Values were considered statistically significant when *P* < .05 (2‐tailed test).

## 3 RESULTS

### 3.1 Microbial diversity does not discriminate between SARI hospitalized patients

Disease outcome for the 12 SARI hospitalized patients who were sampled for this study varies, with most deaths (4/5) occurring in the IAV‐positive group and only 1 in the non‐IAV group (Table 1). All 6 IAV‐positive patients were infected with the 2009 pandemic H1N1 strain (A/H1N1pdm09) (Table 1).^20^ All 6 non‐IAV patients received treatment to alleviate symptoms of respiratory illness during hospitalization once samples tested negative for respiratory viruses.

**Table 1:** Demographic and clinical characteristics for influenza diagnosis and disease outcome of severe acute respiratory infection hospitalized patients

Patient^a^	Age	Gender	IAV	Outcome
IP1	38	Female	Positive	Death
IP2	29	Female	Positive	Cure
IP3	43	Male	Positive	Cure
IP4	2	Female	Positive	Death
IP5	52	Female	Positive	Death
IP6	28	Female	Positive	Death
IN1	47	Female	Negative	Death
IN2	54	Male	Negative	Cure
IN3	60	Male	Negative	Cure
IN4	<1^b^	Female	Negative	Cure
IN5	<1^c^	Female	Negative	Cure
IN6	34	Female	Negative	Cure

Abbreviation: IAV, influenza A virus.

a Patient IDs are IP for “influenza positive” and IN for “influenza negative.”

b Patient was 7 months old.

c Patient was 3 months old.

Microbial diversity was compared between the IAV and non‐IAV SARI patients to determine if the URT microbiome was altered under influenza infection. A total of 673 186 good quality sequence reads, with a mean length of 187 bp from the amplified V4 region of the 16S rRNA gene, were obtained, with an average of 56 000 sequences per sample. Clustering led to the identification of 2543 total OTUs from the 12 samples.

To determine if ecological features could differentiate the IAV patients and non‐IAV patients, we measured the Shannon index for bacterial diversity, the Chao1 index for bacterial richness, and the Simpson_e index for evenness. An Equitability measure was also applied to determine the distribution of OTUs observed (Table 2). No statistical differences were observed for Shannon (Mann‐Whitney *U* test; *P* = 1.000), Chao1 (Mann‐Whitney *U* test; *P* = .631), Simpson_e (Mann‐Whitney *U* test; *P* = .522), or Equitability (Mann‐Whitney *U* test; *P* = .749) between samples of IAV patients and non‐IAV patients.

**Table 2:** Ecological measures of the bacterial community from the nasopharynx of patients with severe acute respiratory infection

	Patient	#OTU^a^	Shannon^b^	Chao1^c^	Simpson_e^d^	Equitability^e^
IAV patients	IP1	189	4.732	212.214	0.066	0.676
	IP2	217	4.654	244.067	0.048	0.641
	IP3	90	2.883	102.750	0.043	0.501
	IP4	316	5.382	350.182	0.053	0.703
	IP5	123	2.645	139.667	0.028	0.435
	IP6	317	5.570	349.523	0.077	0.733
Non‐IAV patients	IN1	49	1.698	142.000	0.047	0.306
	IN2	203	5.375	242.667	0.102	0.749
	IN3	268	4.817	297.129	0.058	0.663
	IN4	186	3.832	202.235	0.032	0.553
	IN5	244	4.608	309.045	0.049	0.647
	IN6	341	6.241	378.500	0.084	0.758

Abbreviations: IAV, influenza A virus; IN, influenza negative; IP, influenza positive.

a Operational taxonomic units.

b Diversity index.

c Richness index.

d Evenness index.

e Pielou index.

### 3.2 The genus Pseudomonas is associated with non‐IAV hospitalized patients

The taxonomic classification of the sequences for these 12 samples revealed the nasopharynx to be colonized by 9 bacterial phyla, albeit not all simultaneously. A comparison between IAV patients and non‐IAV SARI patients showed significant differences in the frequencies of Proteobacteria and Firmicutes.

**Figure 1:**
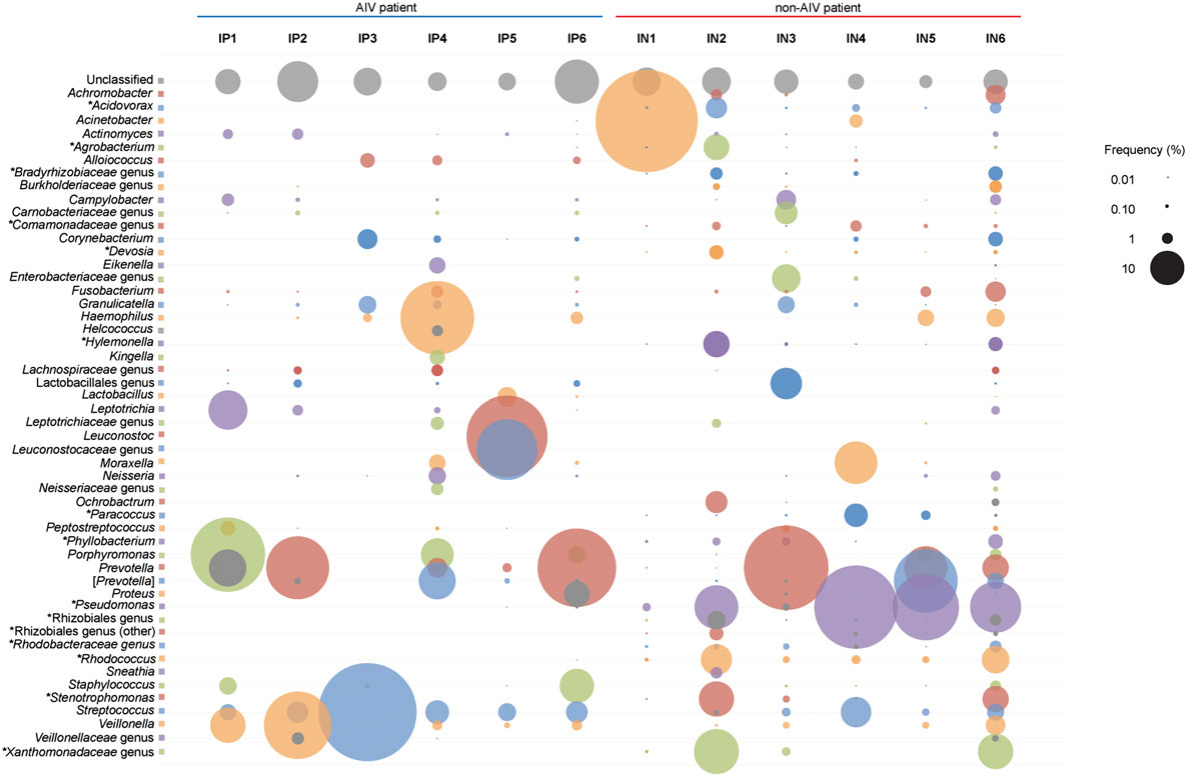
Relative abundance of operational taxonomic units across samples. Circle sizes represent the relative abundance of each operational taxonomic unit present at a frequency above 1%. Genera with frequency differences between groups are marked with an asterisk (*). Abbreviations: IAV, influenza A virus; IN, influenza negative; IP, influenza positive

At 97% similarity, the sequences matched 110 different OTUs, from which 52 (47.2%) had frequencies above 1% of the total reads. Ten of the 12 samples had at least one‐third of the total reads overrepresented by 1 genus. In general, the most abundant bacterial genera found across samples were *Prevotella* (at an average relative abundance of 15.8%), *Pseudomonas* (11.7%), and *Streptococcus* (9.5%). Significant differences between IAV and non‐IAV groups were seen for 15 genera (Figure 1). At all taxonomic levels, the sequences that could not be classified to known taxa ranged from 1.4% to 16.9% of the total reads, and no significant differences were observed.

Interestingly, the bacterial genus *Pseudomonas* appeared to be absent—or present at a very low relative abundance (0.01% and 0.06% in 2/6)—in samples from IAV patients, while it was present at a high relative abundance in 5 of the 6 samples from non‐IAV patients (Figure 1). The genus *Streptococcus* was present in nearly all samples (IN1 is the exception), although relative abundance seemed to be higher in the IAV group. No significant difference was observed (Mann‐Whitney *U* test; *P* = .055) for the relative frequency of the genus *Streptococcus* when groups were compared.

**Figure 2:**
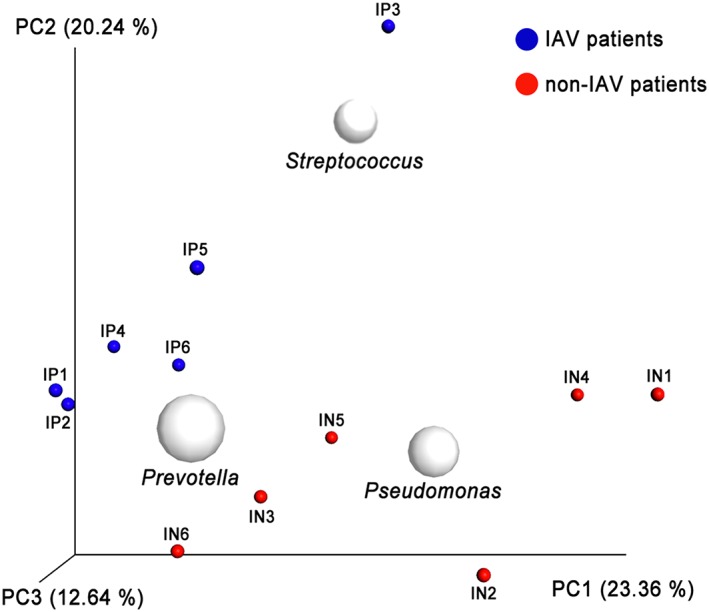
Clustering of samples by similarity and diversity via PCoA. Clustering of patient samples by bacterial diversity using unweighted UniFrac. White circles represent the centering of the 3 most frequent genera plotted using BiPlot on the basis of summarize taxa table generated on QIIME v1.8.0. Red dots represent non‐IAV patient samples; blue dots represent IAV patient samples. PCoA 3D plots were visualized using EMPeror. Abbreviations: IAV, influenza A virus; IN, influenza negative; IP, influenza positive; PCoA, principal coordinate analysis

The pairwise value using unweighted UniFrac and PCoA to cluster samples based on sequence information revealed an association between the bacterial community and the IAV infection profile (Figure 2). Individual samples fell into 2 separate clusters, corresponding to IAV and non‐IAV, suggesting that the bacterial community in the nasopharynx of both groups of patients is different. When PCoA plotted the 3 most abundant genera, results indicated that the genus *Pseudomonas* (Phylum Proteobacteria) was associated with non‐IAV patients. Conversely, on the basis of the difference of abundance observed between groups, the genus *Streptococcus* (Phylum Firmicutes) was associated with IAV patients (Figure 2).

## 4 DISCUSSION

This was a pilot study to verify whether differences existed in the bacterial community of nasopharyngeal samples collected from SARI hospitalized patients with and without IAV H1N1pdm09. Once the microbiome of the URT of SARI patients either infected or noninfected by IAV is known, patterns of microbial communities can be identified, and personal therapeutic strategies can be planned, including antibiotic use for patients with severe disease. This information can be useful to reduce the number of hospitalizations due to suspected IAV infections and to undertake adequate treatment measures to control bacterial secondary disease spreading in health units.

In this study, both IAV patients and non‐IAV patients exhibited a great bacterial diversity in the nasopharynx, and the average number of OTUs obtained was very similar between the IAV (1252) and non‐IAV (1291) patients. Each group had 1 sample with a low abundance of OTUs (IP3 in IAV group and IN1 in non‐IAV group), meaning a lower bacterial richness and dominance of specific bacteria.

The major contributor to the high relative abundance of Firmicutes in the IAV group was the genus *Streptococcus*, which was present in nearly all samples but in different relative abundance between the IAV and non‐IAV groups. However, this difference was not significant (Mann‐Whitney *U* test; *P* = .055), and a larger sample set would be required to confirm this trend. *Streptococcus* has been reported as an important agent of secondary bacterial pneumonia in IAV patients^29^ and benefits from co‐occurrence with IAV, due to the modulation of the host innate immune response^17^; it is also important in controlling colonization (negative association of cocolonization) of the URT by other pathogenic species such as *Staphylococcus*.^10^ These positive and negative associations between OTUs on high frequencies on URT, such as *Streptococcus*, and those OTUs present on low frequencies should be appreciated in a cohort with a larger number of samples.^30^

Nasal carriage of *Staphylococcus* has been shown as a significant risk factor for bacterial secondary infection in patients infected with IAV.^31^, ^32^ However, our data showed a low frequency of *Staphylococcus* on samples of IAV group (average, 2.17%) and non‐IAV group (average, 0.18%). Only 3 of 12 samples (IP1, IP6, and IN6) had more than 1% of frequency, and association to IAV patients was not observed for this bacterial genus. Langevin et al^9^ also observed a low frequency of *Staphylococcus* on nasopharynx samples of patients with severe influenza. Thus, despite the fact that pathogenic bacteria (such as *Staphylococcus*) are frequently present in the nasopharynx of patients, infection of URT depends on a complex interplay involving bacteria‐virus‐host interaction.^10^ These competitive interspecies interactions based on negative and positive association still needs to be better understood.

An association between IAV and *Pseudomonas* was previously reported, suggesting that IAV infection may facilitate the establishment of this pathogenic bacterium in the lower respiratory tract.^33^ In our study, however, we did not find evidence for such association. In fact, the genus *Pseudomonas* was identified in only 2 of 6 IAV samples (IP5 and IP6) and at a very low relative abundance (less than 1%), whereas it was found at a high relative abundance in all non‐IAV samples. *Pseudomonas* is a bacterial genus related to several human infections, and it has been considered an opportunistic pathogen present in the respiratory tract of humans.^34^ Some species of this genus are able to produce a biofilm and express flagellum protein as well as several other adhesins, such as pili. These characteristics are important for colonization and adhesion to mucins, glycoproteins found in airway mucus.^35^

*Pseudomonas* has been shown to induce host expression of *MUC2* and *MUC5AC*, contributing to excessive mucus production in the lungs. Mucins, the main content of mucus, are rich in sialic acids (SAs). Influenza A virus enters the host cell by binding to receptors that contain SA in the cell membrane; the release of IAV from the cell membrane, in turn, requires hydrolysis of the SA linkage by the neuraminidase.^1^ Interestingly, mucins seem to play a protective role against influenza virus infection in mice overexpressing Muc5ac.^36^ Severe disease by influenza virus might be prevented by the presence of great amounts of mucins on the respiratory tract. The increase of mucus stimulated by the presence of *Pseudomonas* on the respiratory microbiome would make mucins important competitors for the viral hemagglutinin, resulting in the requirement of highly efficient viral neuraminidase activity for virion release to infect new cells.^36^ Other mechanisms of negative association may be involved on IAV and *Pseudomonas* interaction. Recently, a study has suggested that bacterial lipopolysaccharide can interact directly with and destabilize influenza virion.^37^ That possibility is a new approach to be considered on the fight against severe influenza infection.

Studies of the microbiome using samples from the URT of patients hospitalized with SARI require strategies to minimize bias that may exist, considering the short term of clinical course that characterizes influenza infection. The virus reaches the replication peak 48 hours post infection in the nasopharynx, and then viral replication decreases slowly during the 6 subsequent days until it reaches a viral load that is too low to be detected.^38^ Thus, failure in the detection of IAV in clinical samples can be avoided if nasopharynx samples are promptly collected once the patient is hospitalized with SARI. In this study, most of the hospitalized patients exhibited a unique combination of comorbidities as well as behaviors such as smoking, which turned patients ineligible for the study. Because of those, the present study has a limited number of samples, which hampered multivariate analyses (age, sex, and outcome).

Our results suggest a trend that would need to be confirmed with a larger number of specimens to determine whether a specific microbiota in the URT that is associated with severity of disease indeed exists, such that could be predictive of poor outcomes in patients infected with influenza. On the basis of our findings, we suggest that the presence of *Streptococcus* is not necessarily indicative of poor outcome in IAV patients, but that shifts in its relative abundance, or even concomitance of its presence with the presence or absence of other specific species, may be. Our findings also suggest that in patients with SARI, *Pseudomonas* and IAV are not always found in association.

## FUNDING

This study was supported by the Graduate Program in Pathology of UFCSPA, the Coordenação de Aperfeiçoamento de Pessoal de Nível Superior (CAPES‐MEC, Brazil), the Fundação de Amparo à Pesquisa do Estado do Rio Grande do Sul (FAPERGS, Edital 02/2014–PqG no. 2282–2551/14–5), and the Conselho Nacional de Desenvolvimento Científico e Tecnológico (Fellowship 311309/2012‐7 held by A.B.G.V.).

## CONFLICT OF INTEREST

All authors declare no conflicts of interest.

## AUTHOR CONTRIBUTIONS

Conceptualization: Luiz Gustavo dos Anjos Borges, Adriana Giongo, Ana Beatriz Gorini da Veiga

Data curation: Luiz Gustavo dos Anjos Borges, Adriana Giongo

Formal analysis: Luiz Gustavo dos Anjos Borges, Leandro de Mattos Pereira, Fernanda J. Trindade

Funding acquisition: Adriana Giongo, Ana Beatriz Gorini da Veiga

Investigation: Luiz Gustavo dos Anjos Borges, Tatiana Schaffer Gregianini, Adriana Giongo, Ana Beatriz Gorini da Veiga

Methodology: Luiz Gustavo dos Anjos Borges, Leandro de Mattos Pereira, Fernanda J. Trindade, Tatiana Schaffer Gregianini, Adriana Giongo, Ana Beatriz Gorini da Veiga

Project administration: Fabrício Souza Campos, Adriana Giongo, Ana Beatriz Gorini da Veiga

Resources: Fabrício Souza Campos, Tatiana Schaffer Gregianini, Adriana Giongo

Supervision: Ana Beatriz Gorini da Veiga

Validation: Luiz Gustavo dos Anjos Borges, Adriana Giongo

Visualization: Luiz Gustavo dos Anjos Borges, Adriana Giongo

Writing–original draft preparation: Luiz Gustavo dos Anjos Borges

Writing–review and editing: Ana Beatriz Gorini da Veiga, Elodie Ghedin
